# Analysis of amino acids in *Mucuna pruriens* supplements using hydrophilic interaction liquid chromatography-tandem mass spectrometry

**DOI:** 10.1007/s00726-025-03491-0

**Published:** 2026-01-23

**Authors:** Connor R. Phillips, Jake P. Violi, David P. Bishop, Kenneth J. Rodgers

**Affiliations:** 1 School of Life Sciences, Faculty of Science, University of Technology Sydney, Sydney, NSW Australia; 2 Hyphenated Mass Spectrometry Laboratory (HyMaS), University of Technology Sydney, Sydney, NSW Australia; 3 School of Chemistry, University of New South Wales, Sydney, NSW Australia; 4 School of Mathematical and Physical Sciences, University of Technology Sydney, Sydney, NSW Australia

**Keywords:** L-DOPA, Parkinson’s disease, Non-protein amino acid, Mass spectrometry, Mucuna pruriens

## Abstract

*Mucuna pruriens* (MP), or velvet bean, has been used as an alternative medicine in India for over 4500 years, predominantly due to the natural abundance of the non-protein amino acid L-3,4-dihydroxyphenylalanine (L-DOPA). L-DOPA (levodopa) is used to treat Parkinson’s disease (PD), a condition in which the progressive loss of dopaminergic neurons causes dopamine deficiency and impaired motor function. Although L-DOPA increases dopamine synthesis in the remaining dopaminergic neurones in the PD brain, the rate of disease progression appears to remain unchanged. Some studies have demonstrated improved outcomes in patients taking MP preparations compared to those undergoing traditional L-DOPA therapy. There is evidence that the canonical amino acids and L-DOPA precursors L-phenylalanine (L-Phe) and L-tyrosine (L-Tyr) can increase dopamine synthesis and also protect against the mistaken incorporation of L-DOPA into proteins during protein synthesis. The current study developed and validated a sensitive HILIC–TQMS method for the quantification of L-DOPA and related amino acids in MP preparations. Analysis revealed that L-DOPA levels were 66.2% to 82.7% of the values reported by manufacturers. Tyr and Phe were present in both free and protein bound forms in all 5 preparations analysed, potentially offering protection against the mistaken incorporation of L-DOPA into proteins and promoting increased dopamine synthesis. These findings suggest that the additional reported benefits of MP supplements for PD treatment might, in part, be attributable to the presence of these amino acids, further supporting the need to investigate the administration of L-DOPA and its cognate amino acid in symptomatic treatment of PD.

## Introduction

**Fig. 1:**
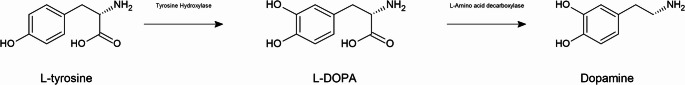
Schematic representation of the dopamine synthesis pathway

*Mucuna pruriens* (MP), also known as velvet bean, is a species of climbing legume belonging to the Fabaceae family and is native to many tropical and subtropical areas of the world (Cassani et al. 2016). MP seeds, which contain the dopamine precursor 3,4-dihydroxyphenylalanine (L-DOPA, levodopa), have been used to treat Parkinson’s disease (PD) in India for over 4500 years (Kavitha and Thangamani 2024). Dopaminergic neurons of the substantia nigra pars compacta are the main source of dopamine in the mammalian central nervous system but their progressive loss in PD results in dopamine deficiency and impacts voluntary movement and a broad array of behavioural processes (Chinta and Andersen 2005). Treatment with L-DOPA increases the synthesis of the neurotransmitter dopamine in the remaining dopaminergic neurones (Fig. 1). Additional properties of the MP plant further support its medicinal use such as its antioxidant (Dimitry et al. 2022), anti-inflammatory (Han et al. 2022) and hypoglycaemic effects (Bhaskar et al. 2008) (reviewed by Rai (Rai et al. 2020)). L-DOPA was first identified and extracted from MP in 1937 (Damodaran and Ramaswamy 1937), leading to its use in complementary medicine primarily for the treatment of PD as a ‘natural’ alternative to pharmaceutical L-DOPA. In addition, powder made from ground MP seeds is used in regions of the world where affordability makes pharmaceutical L-DOPA more difficult to acquire (Cassani et al. 2016).

There is anecdotal evidence that the protein amino acid L-tyrosine (L-Tyr) was used as an early treatment for PD; however, its use might have been discontinued based on the false assumption that a decrease in tyrosine hydroxylase (TH) activity was the primary deficit in the PD brain (Martin 1971) (Fig. 1) rather than the loss of dopaminergic neurones (Fearnley and Lees 1991). L-Tyr is the physiological precursor of L-DOPA (Fig. 1) and its administration can increase the rate of catechol synthesis (Wurtman et al. 1974) stimulating dopamine synthesis in the remaining dopaminergic neurones in the PD brain. L-Tyr hydroxylation is the rate-limiting step in catecholamine synthesis, and it was assumed that TH is normally close to full saturation with its substrate L-Tyr and thus L-DOPA synthesis will not be increased by increasing L-Tyr (Fitzpatrick 1999; Kaufman 1995). When this was directly examined in vivo however extracellular L-DOPA levels in the striatum and medial prefrontal cortex increased up to 250% and 300% of control levels respectively in response to increasing L-Tyr concentrations, leading the authors to conclude that TH is not near full saturation with L-Tyr under normal conditions (Brodnik et al. 2012).

Oral administration of L-Tyr significantly increased plasma Tyr levels (2–3 fold) even in non-fasted PD patients (Melamed et al. 1980), and led to elevated levels of Tyr and homovanillic acid, a major metabolite of dopamine, in the cerebrospinal fluid (Growdon et al. 1982). Investigation into the use of L-Tyr in PD ceased with the introduction of L-DOPA, however it remains possible that PD patients would obtain significant benefit from oral L-Tyr. It is possible that in addition to L-DOPA, MP preparations contain L-Tyr and its precursor phenylalanine (L-Phe) which could provide additional therapeutic benefit to PD patients by increasing dopamine production (Wurtman et al. 1974).

One limitation of L-DOPA therapy is the development of dyskinesias and fluctuations in motor response (Rua Rafael et al. 2016). The mechanisms of L-DOPA-induced motor complications are still poorly understood, with research suggesting the pulsatile nature, or the non-continuous availability of dopamine produced from exogenous L-DOPA therapy, alters post-synaptic dopamine receptors (Kulkarni et al. 2025). Another potential drawback of L-DOPA is its ability to mimic canonical amino acids. Studies have shown that L-DOPA can be mistakenly incorporated into proteins in place of L-Tyr (Chan et al. 2012; Rodgers et al. 2002, 2006) and Phe (Moor et al. 2011) and, as is the case for some other ‘proteinogenic’ non-protein amino acids (NPAAs), can evade mechanisms of quality control (Mohler and Ibba 2017; Rodgers 2014). The ability of L-DOPA to mimic the protein amino acids L-Tyr and L-Phe can lead to the synthesis on aberrant aggregate-prone proteins (Dunlop et al. 2009, 2011), aggregate accumulation, and mitochondrial damage (Giannopoulos et al. 2019). Consistent with this, L-DOPA-containing proteins are elevated in the motor cortex, occipital cortex and substantia nigra of patients undergoing prolonged L-DOPA therapy relative to control patients (Chan et al. 2012) and this might contribute to some of the motor complications experienced following chronic L-DOPA exposure. Co-administration of the ‘parent’ cognate protein amino acids can be protective against proteinogenic NPAAs due to the specificity of the tRNA synthetase being significantly higher for the cognate amino acids (Rodgers and Shiozawa 2008). Any L-Tyr and L-Phe present in MP preparations and co-administered with L-DOPA could therefore provide some protection against the generation of toxic L-DOPA-containing proteins.

When doses of MP were adjusted to achieve equivalent DOPA plasma levels to those of pharmaceutical DOPA administered with an amino acid decarboxylase inhibitor (AADCI), patients receiving the MP preparation showed improvement in motor symptoms, a longer duration of ‘on’ state and reduced dyskinesias (Cassani et al. 2016; Katzenschlager et al. 2004). These findings supported studies in primates (Lieu et al. 2012) and rats (Kasture et al. 2009; Lieu et al. 2010), which reported that MP exhibits better anti-parkinsonian activity than conventional DOPA therapy, with lower risk of dyskinesias and a better tolerability profile (Cilia et al. 2017; Lieu et al. 2010). A recently published review of clinical trials on the use of MP in PD, identified only 5 trials that met quality criteria (Hammoud et al. 2025). Of these, one study was rated as high quality, one as having some concerns, and three as low quality. Nevertheless, the findings consistently demonstrated improvements in PD symptoms and therapy-related complications, including a shorter time to reach the ‘on’ disease state, prolonged duration of this state, and fewer adverse events, with no dyskinesia reported (Hammoud et al. 2025). However, current clinical evidence on the efficacy of MP preparations remains limited, and further high-quality trials are needed to confirm their efficacy and safety.

The most widely used analytical method to detect L-DOPA in plant matrices is high-performance liquid chromatography (HPLC) coupled with diode array detection (DAD) at 280 nm (HPLC-UV) (Tesoro et al. 2022). However, this method lacks selectivity because other compounds can absorb at the same wavelength. Detection by mass spectrometry (MS), on the other hand, can provide a unique and unambiguous identification with increased sensitivity. In the present studies, we developed and validated a sensitive HILIC-TQMS method for the analysis of L-DOPA in both free and protein-bound forms in MP seed preparations. By targeting a previously undescribed L-DOPA-acetonitrile (ACN) adduct (Violi et al. 2021) we achieved lower limits of detection (LODs) and lower limits of quantification (LOQs) than those reported in other published methods (Tesoro et al. 2023). The method was also used to quantify the Tyr and Phe content in MP preparations, as well as the Phe oxidation products *ortho*-Tyr and *meta*-Tyr.

## Materials and methods

### Materials

L-DOPA, L-DOPA-(phenyl-d_3_), L-Tyr, L-Phe, DL-*o*-tyrosine (*ortho*-tyrosine), and L-*meta*-tyrosine standards were purchased from Sigma-Aldrich (Castle Hill, NSW, Australia). LiChrosolv^®^ hypergrade acetonitrile (ACN) for LC–MS, ammonium formate, LC–MS LiChropur™ formic acid, and trichloroacetic acid were purchased from Sigma-Aldrich (Castle Hill, NSW, Australia). All 5 formulations of *Mucuna pruriens* (MP) supplements were purchased from the online vendor iHerb (Australia).

### Sample preparation

Five MP preparations were analysed and details of the composition and recommended dosage as a general use dietary supplement (per manufacturer guidelines) are presented in Table 1. For each supplement, three dose units (caplets/capsules) were ground into a fine powder using a mortar and pestle to generate a pooled representative sample of each. Samples that were externally coated were removed from their casings using stainless steel scissors before weighing. From these pooled samples, 50 mg of dry powder of each sample was weighed in triplicate. Each sample was suspended in 1 mL of 10% TCA in water, probe sonicated at 50% power for 30 s twice, maintaining samples on ice between cycles before being left in the refrigerator overnight to allow protein precipitation. Samples were spun at 15,000 x g at 4 °C for 15 min with subsequent removal of the supernatant. A further 300µL of 10% TCA was added to the pellet for resuspension, with centrifugation and supernatant removal as previously described in duplicate. The supernatant containing the free fraction was lyophilised and reconstituted in 200 µL of 20 mM HCl and stored in a − 80 °C freezer prior to analysis. The pellet containing the bound fraction was transferred to glass shell vials in 400 µL of 100% acetone, centrifuged for 5 min at 5000x*g* and dried in a centrifugal evaporator. Samples were hydrolysed in glass vials containing 1 mL of 6 M HCl, 50 µL mercaptoacetic acid and 10 µL of phenol. Air in the vials was exchanged for nitrogen gas three times and then a vacuum was drawn and the hydrolysis vessels sealed and incubated in an oven set to 110 °C for 16 h. Hydrolysed samples were reconstituted in 200 µL of 20 mM HCl, centrifuged at 5000x*g* for 2 min, transferred to 0.2 µM membrane filters and spun for a further 30 min at 5000x*g*. The hydrolysed bound fraction samples were then stored in a − 80 °C freezer before analysis. All samples and standards were diluted 1 in 10 into ACN as to minimise discrepancies between sample and mobile phase composition.

**Table 1:** Summary of the *Mucuna pruriens* (MP) samples analysed based on data provided by the manufacturers

Supplement	Dosage and Form	Ingredient List (/serving size)	L-DOPA/ day (mg)
*A*	1–6 capsules/day	*Mucuna pruriens* seed extract (166 mg), maltodextrin, hydroxypropyl methylcellulose, magnesium stearate and silica	100–600
*B*	2 capsules/day	*Mucuna pruriens* seed extract (800 mg), hypromellose, microcrystalline cellulose and stearic acid	120
*C*	1 capsule/day	*Mucuna pruriens* seed extract (200 mg), modified cellulose	30
*D*	2 capsules/day	*Mucuna pruriens* seed extract (333 mg), alpha galactidase (5 mg), maltodextrin, rice flour, vegetable cellulose, magnesium stearate, enteric coating	100
*E*	1 caplet/day	*Mucuna pruriens* seed extract (250 mg), *Mucuna pruriens* stem extract (350 mg)	N/A

### Hydrophilic interaction liquid Chromatography-Tandem mass spectrometry (HILIC-TQMS)

**Fig. 2:**
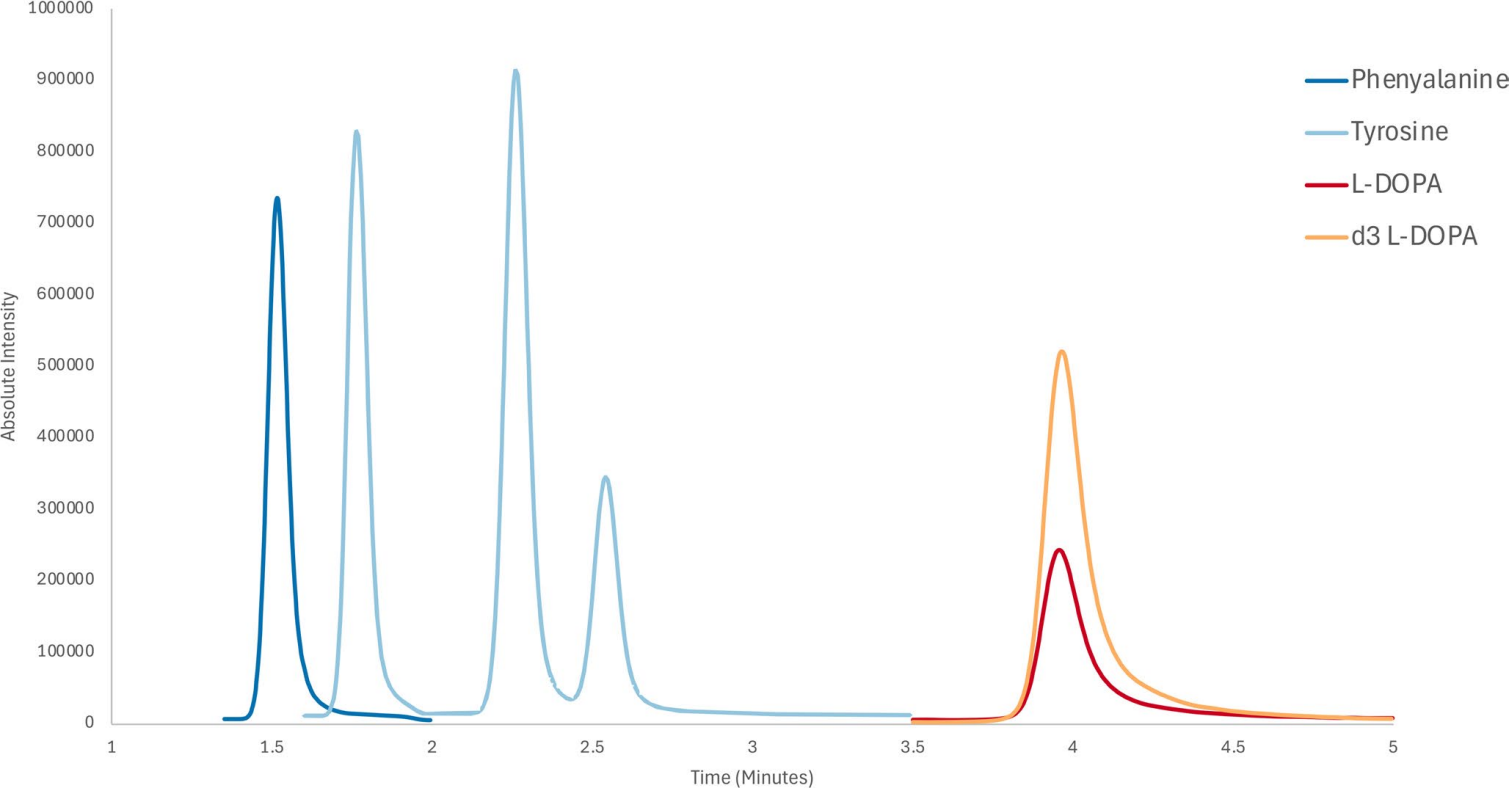
TIC chromatogram of analytes. Tyrosine isomer peaks elute in the order; 1) ortho-tyrosine, 2) meta-tyrosine, and 3) para-tyrosine

Chromatographic separation of metabolites was performed using the Shimadzu Nexera X2 UHPLC system with analyte detection performed on a Shimadzu 8060 triple quadrupole mass spectrometer (TQMS). A Waters™ ACQUITY UPLC BEH Amide column (2.1 × 100 mm, 1.7 μm particle size) was used for separation with a flow rate of 0.8 mL/min and column oven temperature set to 30 °C. The method utilised a binary pump system with Solvent A consisting of 10 mM ammonium formate in ultrapure water + 0.5% formic acid, and Solvent B consisting 10 mM ammonium formate in a 90:10 mix of acetonitrile: ultrapure water + 0.5% formic acid. The solvent elution for separation is isocratic from 0.00 min to 5.00 min at 97.5% Solvent B, before a wash period from 5.01 min to 7.00 min at 80% Solvent B and a re-equilibration period from 7.01 min to 15.00 min at 97.5% Solvent B. The injection volume for sample was set to 1 µL. The Shimadzu 8060 TQMS was equipped with an electrospray ionisation (ESI) source set to positive ionisation mode with the following source parameters: 0.1 kV interface voltage, 300 °C interface temperature, 225 °C desolvation line (DL) temperature, 400 °C heat block temperature, 3 L/min nebulising gas flow, 17 L/min heating gas flow and 3 L/min drying gas flow. Nitrogen was used for drying, heating and nebulising gas, while argon was used for the collision gas. During method development, multiple reaction monitoring (MRM) ion transitions were established for each compound using a combination of protonated ([M + H]^+^) and ACN adduct ([M + H + ACH]^+^) mass-to-charges (m/z) as previously described by Violi (Violi et al. 2021) (Table 2). MRMs were segmented based on elution window, with each metabolite having its own window except for Tyr (*para*-Tyr) which was separated from its structural isomers (*meta-Tyr* and *ortho*-Tyr) within the same window (Fig. 2).

**Table 2:** Multiple reaction monitoring (MRM) transitions and settings for compounds of interest using both protonated and acetonitrile (ACN) adduct masses. Quantifying ion is denoted by an asterisk (*). Tyrosine MRMs allow for resolution of all three isomers

Compound	Dwell Time (msec)	Collision Energy (eV)	Retention Time (mins)	MRM Transitions (m/z)
L-Phenylalanine	25	− 12.4 − 12.4 − 23.4	1.57	*207.1 → 120.1 166.2 → 120.1 → 103.1
L-Tyrosine	25	− 14.7 − 45.1 − 14.7	2.63	*223.1 → 136.2 → 91.05 182.2 → 136.2
L-DOPA	45	− 18.9 − 21.8 − 16.9	4.15	*239.2 → 181.1 → 152.1 → 139.1

### Method validation and data analysis

An 8-point calibration curve was created for the three analytes of interest (1, 10, 25, 50, 100, 250, 500, and 1000 ng/mL). Repeatability was determined from calculation of %RSD from 5 repeat injections from one point in the standard curve. Limits of detection (LOD) and limits of quantification (LOQ) were determined by a signal to noise (S/N) ratio of 3.3 and 10 respectively, from spiked sample matrix (pooled sample). Method validation was performed by determination of the linear range, coefficient of determination (R^2^), repeatability (%RSD) determined from peak area, limit of detection (LOD) and limit of quantification (LOQ) for the five analytes by using a representative pooled sample matrix and standard addition (Table 3). Deuterated L-DOPA (L-DOPA-d_3_) was used as an internal standard at 100 ng/mL for normalisation of instrument response and was added during the 1 in 10 dilutions of sample into ACN before analysis.

**Table 3:** Summary of validation data for compounds of interest. limit of detection (LOD) and limit of quantification (LOQ) values were determined from matrix spiked samples

Compound	Linear Range (ng/mL)	Regression (*R*^2^)	Repeatability (%RSD)	LOD (ng/mL)	LOQ (ng/mL)
L-Phenylalanine	1-1000	0.99	4.04	0.10	0.31
L-Tyrosine	1-1000	0.99	4.19	0.08	0.24
*L-Meta*-Tyrosine	1-1000	0.99	5.36	0.05	0.14
*DL-Ortho*-Tyrosine	1-1000	0.99	4.87	0.07	0.21
L-DOPA	1-1000	0.99	5.48	0.20	0.60

All data analysis and method validation were conducted using Shimadzu LabSolutions and GraphPad Prism V8.0.1.

## Results

### Method validation

Analyte response for all five analytes (L-Phe, L-DOPA, (*para*-) L-Tyr, *L-meta-Tyr* and DL-*ortho*-Tyr) was demonstrated to be linear from 1 ng/mL to 1000 ng/mL. The repeatability of analyte response, as determined by integrated peak area, was under the acceptable threshold of 10%, with none exceeding a percentage relative standard deviation of 5.5%. Spikes of all 5 analytes in a representative pooled matrix of the 5 supplements analysed demonstrated LODs and LOQs of below 0.2 ng/mL and 0.6 ng/mL respectively (Table 3). The percentage recovery of all analytes spiked at 100 ng/mL in representative pooled matrix samples was above 90% for all analytes (Table 4). A comparison of the signal-to-noise ratio (S/N) of the protonated MRM (198.10 ◊ 152.00 *m/z*) against the ACN adduct MRM (239.100 → 152.00 *m/z*) for the analyte L-DOPA showed the ACN adduct MRM to have an approximate 4.5-fold increase in S/N ratio.

**Table 4:** Percentage recovery of 100 ng/mL analyte spikes in representative pooled matrix (*n* = 3)

Compound	Percentage Recovery (%)
L-Phenylalanine	91.33
L-Tyrosine	99.15
*L-Meta*-Tyrosine	97.32
*DL-Ortho*-Tyrosine	94.61
L-DOPA	93.78

### Quantification of L-DOPA

The total amount of L-DOPA, both free and protein bound, in the dosage form for each supplement was determined using the developed method and compared to the amount of L-DOPA reported by the manufacturer, except for supplement E where the manufacturer did not report the L-DOPA content (Table 5). The highest amount of L-DOPA found among the supplements was 70.1 mg and accounted for 70.1% of that reported by the manufacturer. The lowest amount found amongst the supplements was 15.74 mg in the supplement where the manufacturer did not disclose the amount. The L-DOPA content found in the supplements relative to that reported by the manufacturers therefore ranged from 66.2% to 82.7%. The HILIC–TQMS method employed is unable to distinguish between the L and D-isomers of amino acids; however, D-isomers of the amino acids measured in the present studies have never been found in plants (Kolukisaoglu 2020).

**Table 5:** Amount of L-DOPA found in samples relative to manufacturer reported values per dosage denomination (tablet/capsule) ± standard deviation (*n* = 3)

Supplement	Free L-DOPA (mg)	Bound L-DOPA (µg)	Total L-DOPA (mg)	Reported L-DOPA (mg)	% of Reported
*Supp A*	70.1 ± 0.59	0.09 ± 0.02	70.1	100	70.1
*Supp B*	39.7 ± 6.86	0.12 ± 0.01	39.7	60	66.2
*Supp C*	24.8 ± 0.37	0.80 ± 0.17	24.8	30	82.7
*Supp D*	40.9 ± 2.56	0.07 ± 0.02	40.9	50	81.7
*Supp E*	15.7 ± 2.37	0.08 ± 0.01	15.7	N/A	N/A

### Quantification of phenylalanine and tyrosine

The amount of Phe and Tyr in both the protein bound and free fraction of the supplements was determined (Table 6). All supplements analysed were found to contain Phe, Tyr and L-DOPA in both the free and bound forms. Tyr occurred in the highest amounts in the free fraction for all supplements analysed, with Phe predominantly concentrated in the free fraction except for supplements B and D where it occurred predominantly in the bound fraction. L-DOPA occurred predominantly in the free fraction. No *ortho*-tyrosine or *meta*-tyrosine were detected in any of the free and hydrolysed samples analysed.

**Table 6:** Amount of phenylalanine (Phe) and tyrosine (Tyr) in each dosage form (caplet/capsule) for the *Mucuna pruriens* supplements showing the free (TCA soluble fraction), bound (protein hydrolysed fraction) ± standard deviation (*n* = 3)

Supplement	Free Phe (µg)	Bound Phe (µg)	Free Tyr (µg)	Bound Tyr (µg)
*Supp A*	39.59 ± 0.20	8.78 ± 0.65	44.21 ± 3.58	3.15 ± 0.57
*Supp B*	111.48 ± 13.28	135.34 ± 2.16	194.77 ± 40.57	13.46 ± 1.06
*Supp C*	31.64 ± 1.00	0.59 ± 0.16	17.63 ± 1.25	0.21 ± 0.05
*Supp D*	95.43 ± 2.09	135.16 ± 0.93	223.73 ± 4.04	3.75 ± 0.28
*Supp E*	327.65 ± 25.14	81.93 ± 2.33	451.59 ± 41.49	8.42 ± 2.34

### Amino acid ratios

The total amounts of Phe and Tyr were determined and compared to the total amount of L-DOPA in the preparations (Table 7). Supplement E contained the highest total L-Phe and L-Tyr content, resulting in the highest ratio of Phe and Tyr to L-DOPA amongst the supplements; 2.6% and 2.9% respectively. The lowest total amount of Phe was found in supplement C, however the lowest observed ratio of Phe to L-DOPA was found in supplement A at 0.069%. The lowest total amount of Tyr was once again found in supplement C resulting in a Tyr to L-DOPA ratio of 0.072%, with the lowest observed ratio of Tyr to L-DOPA being found in supplement A (0.068%).

**Table 7:** Total phenylalanine (Phe) and tyrosine (Tyr) and percentage of each amino acid to total L-DOPA

Supplement	Total Phe (µg)	Phe % of L-DOPA	Total Tyr (µg)	Tyr % of L-DOPA
*Supp A*	48.37	0.069	47.37	0.068
*Supp B*	246.82	0.62	208.24	0.52
*Supp C*	32.23	0.13	17.85	0.072
*Supp D*	230.59	0.56	227.47	0.56
*Supp E*	409.59	2.60	460.01	2.92

Generally, most of the amino acids detected were in the free form with, for example, 80% of phenylalanine and 98% of the tyrosine in the free form in supplement E. The only exceptions being Phe in supplements B and D which had marginally more of the amino acid in the bound form (55% and 59% respectively). For 3 of the supplements the L-Tyr and Phe concentrations were very similar, with Tyr to Phe ratios of 0.98, 0.99 and 1.12 (supplements A, B and D respectively).

## Discussion

### Method validation and potential applications

A HILIC-TQMS method was developed for the analysis of L-DOPA, Phe, and the Tyr isomers (*para*, *ortho* and *meta-*Tyr) which was shown to be highly sensitive and suitable for use with plant matrices such as that present in MP supplements. Traditionally, underivatised analysis of amino acids cannot achieve the high sensitivity of the more commonly used derivatisation methods but in this method, we took advantage of the amino acid-ACN adducts first described by Violi and colleagues (Violi et al. 2021). The optimal transitions for both Phe and Tyr were modified by increasing the collision energy to the current values to better align instrument response for the plant matrix (Violi et al. 2021). L-DOPA, *ortho-* and *meta-Tyr* were also added to this original method, and we demonstrated that the ACN adduct also allowed greater detection sensitivity for these analytes, with a 4.5-fold increase in the S/N of the ACN adduct transition relative to the protonated m/z transition. The LOD and LOQ for L-DOPA in the developed method was 0.2 ng/mL and 0.6 ng/mL respectively, providing superior sensitivity to recent non-derivatised reversed phase LC–TQMS methods used for the detection of L-DOPA in plant matrices (Tesoro et al. 2023; Yumoto et al. 2022).

*Ortho*- and *meta-Tyr* which are generated from hydroxy radical attack on the benzyl ring of Phe (Ipson and Fisher 2016; Montagna et al. 2020) were added to the method to increase its utility and to demonstrate separation of these isomers was achieved. The use of 10 mM ammonium formate and 0.5% formic acid for solvent buffering was determined to be optimal for the separation of all analytes without suppression of analyte signal. The use of a 10% aqueous component in solvent B allowed for the solubility and uniform concentration of ammonium formate throughout the gradient changes, minimising concerns of the precipitation of buffers in the system and maintaining the aqueous levels to sustain the water layer necessary for HILIC stationary phase separation. A column oven temperature of 30 °C was chosen to keep column conditions above ambient room temperature allowing consistent chromatographic separation of analytes. The optimal interface voltage for the observation of the ACN adducts of all analytes in this method was 0.1 kV, with subsequent increases in interface voltage decreasing the occurrence of this adduct. This HILIC-TQMS method could be extended to detect other bioactive compounds in MP preparations such as tryptophan (Violi et al. 2021) and 5-hydroxytryptamine (5-HT) (Li et al. 2024). It could also be utilised for the analysis of oxidised proteins, as we successfully separated L-DOPA, *meta*-tyrosine, *ortho*-tyrosine and *para*-tyrosine - all useful markers of protein oxidation in ageing (Ipson and Fisher 2016; Rodgers and Dean 2000; Rodgers and Shiozawa 2008). In addition, this method could be an analytic tool in pathological conditions characterised by excessive hydroxyl radical production, such as the systemic inflammatory response syndrome associated with sepsis, which can give rise to all three Tyr isoforms (Montagna et al. 2020).

### L-DOPA content of *Mucuna pruriens* (MP) preparations

It is known that the L-DOPA content differs between MP plant varieties (Pulikkalpura et al. 2015) and the L-DOPA content advertised by the manufacturer of the commercially available preparations can differ from that reported following independent chemical analysis. One study evaluated the L-DOPA content of 16 commercially available MP supplements and found that it was very inconsistent with the labelling information; the supplements analysed contained between 8.9% and 86.1% of the advertised amounts (Cohen et al. 2022). In that study however a simple reconstitution of the powdered material in water was carried out prior to MS analysis (Cohen et al. 2022). Since the analytes were present in MP seed, a complex matrix, variable extraction recoveries might be expected, so in the present study probe sonication in trichloroacetic acid (TCA) was employed. The supplements analysed contained L-DOPA in quantities ranging from 66.2% to 82.7% of that claimed to be present, which, given the fact that only one extraction method was tested, would appear to be reasonable.

L-DOPA is sensitive to hydroxyl radical attack so some degradation could occur during storage or processing giving rise to quinones (Rodgers and Dean 2000). The TCA extraction method also allowed us to obtain a protein fraction for acid hydrolysis. The addition of phenol and mercaptoacetic acid (thioglycolic acid) to the acid hydrolysis vessel minimises the oxidative degradation commonly observed during conventional acid hydrolysis (Muramoto and Kamiya 1990). L-DOPA is highly susceptible to oxidative modification (Asanuma et al. 2003), thus the presence of both phenol and mercaptoacetic acid are advantageous during the hydrolysis process (Gieseg et al. 1993). To assess sample oxidation, we analysed MP protein samples for the presence of *meta-Tyr* and *ortho*-Tyr both of which are products of hydroxyl radical damage to Phe residues, and stable markers of protein oxidation (Ipson and Fisher 2016; Rodgers and Dean 2000). No *meta-Tyr* and *ortho*-Tyr were detected in the hydrolysed or free fraction of samples, suggesting that there had been no significant hydroxyl radical damage to amino acids or proteins. L-DOPA oxidation during sample storage could have contributed to the differences reported between the L-DOPA content advertised by the manufacturer and what was detectable in MP preparations (Cohen et al. 2022).

### The potential of tyrosine in *Mucuna pruriens* (MP) preparations to be protective

The aim of the present studies, in addition to quantifying L-DOPA, was to determine if the MP preparations contained sufficient Tyr and Phe to improve the efficacy of L-DOPA in PD patients by increasing dopamine synthesis while also reducing adverse effects by preventing L-DOPA incorporation into proteins. All the supplements analysed contained both free and protein-bound forms of Tyr presumably originating from the MP plant material. A Tyr to L-DOPA ratio of only 1 to 100 is sufficient to prevent L-DOPA-induced protein aggregation and mitochondrial damage in human neuroblastoma cells (Chan et al. 2012; Giannopoulos et al. 2019). Supplement E from the current study contained an amount of free Tyr well above that level (a Tyr to L-DOPA ratio of 1 in 34) while three of the other supplements might provide some level of protection (with Tyr to L-DOPA ratios ranging from of 1 in 138 to 1 in 192). Although the Phe concentration in the protein fraction was consistently higher than the Tyr concentration, the MP preparations examined contained a similar amount of total Phe to total Tyr. It is known that mammalian Phe-tRNA synthetase can also be charged by L-DOPA (Moor et al. 2011) so the presence of Phe could offer some protection against L-DOPA insertion into proteins at the Phe site. In addition, 75% of ingested Phe is converted into Tyr by mammalian phenylalanine hydroxylase (Eldsworth and Roth 1997) so could also boost Tyr concentrations in vivo. The impact of these levels of Tyr and Phe on dopamine synthesis is unknown, however based on current knowledge, they could potentially enhance dopamine synthesis (Brodnik et al. 2012). Nevertheless, it remains uncertain if such low levels of Phe and Tyr would have a clinically meaningful effect.

Tyr and Phe can also compete with L-DOPA for intestinal absorption, uptake into cells, and transport across the blood-brain barrier, but the high capacity of these pathways means that they are not very sensitive to concentrations of the amino acids, unlike protein synthesis where the cognate amino acids have a much higher affinity for the tRNA synthetases than L-DOPA (Chan et al. 2012). The presence of dietary large neutral amino acids (LNAAs) such as Phe, Tyr, and tryptophan has little effect on L-DOPA absorption into plasma but can compete for uptake into the brain (Carter et al. 1989). A dietary protein study however reported a clinically significant protein interaction with L-DOPA only in a subset of PD patients (Virmani et al. 2016). The concentrations of Phe and Tyr present in the MP preparations analysed in the present studies would not be expected to significantly affect L-DOPA levels in the brain.

Two studies comparing the pharmacokinetics of MP to pharmaceutical L-DOPA found that the concentration-time profile of plasma L-DOPA derived from MP preparations had two peaks (Boonmongkol et al. 2025; Cilia et al. 2017). The authors interpreted the second peak, which appeared around 90–120 min, as likely attributable to the presence in MP of a natural AADCI but this has not been supported experimentally (Boonmongkol et al. 2025). The second peak could possibly represent the release of protein-incorporated L-DOPA providing a ‘slow-release’ form of L-DOPA, although based on the preparations analysed in the present study this would only account for up to 3% of the total L-DOPA. In addition to Tyr and Phe, MP is rich in bioactive compounds with potential neuroprotective and dopamine-modulating effects, such as 5-hydroxytryptophan (5-HTP) which may influence mood and motor function, and tannins and alkaloids, which could potentially reduce dopamine degradation (Rai et all. 2020). A better understanding of the composition of MP preparations and an understanding of their potential therapeutic benefits could lead to improved drug design for PD.

## Conclusions

The current study developed and validated a sensitive HILIC-TQMS method for the quantification of L-DOPA and related amino acids in MP supplements. The sensitivity of the method was significantly enhanced by detecting ACN-adducts of the amino acids (Violi et al. 2021). Analysis revealed that L-DOPA levels were 66.2% to 82.7% of the values provided by the manufacturers. Tyr and Phe were present in all 5 preparations analysed and might help prevent the mistaken incorporation of L-DOPA into proteins while promoting increased dopamine synthesis. These findings suggest that the additional reported benefits of MP supplements for PD treatment might, in part, be attributable to the presence of these amino acids, reinforcing the need to investigate the combined administration of L-DOPA alongside its cognate amino acid for the symptomatic treatment of PD.

## Data Availability

The data that support the findings of this study are available from the corresponding author, KJR., upon reasonable request.
